# Human Pythiosis

**DOI:** 10.3201/eid1203.051044

**Published:** 2006-03

**Authors:** Jakrapun Pupaibool, Ariya Chindamporn, Kanitha Patarakul, Chusana Suankratay, Wannasri Sindhuphak, Wanla Kulwichit

**Affiliations:** * Chulalongkorn University, Bangkok, Thailand

**Keywords:** pythiosis, arteritis, necrotizing cellulitis, letter

**To the Editor:** Over the past 2 decades, human pythiosis has emerged as an important parafungal disease; Thailand reports the most cases (*1**,**2*). Given the rarity of this infection in humans and the limited attention of researchers to the disease, our understanding of its pathogenesis and other important traits, including its management, await investigation. We report 2 distinct cases.

The first patient was a 63-year-old woman with hemoglobin AEBart's disease, a complex thalassemia/hemoglobinopathy syndrome, which required frequent blood transfusions. Two months before admission, after a major flood and exposure to standing water for weeks, an abnormal sensation developed in her right foot, which progressed to pain and intermittent claudication. Subsequent inability to walk prompted her to seek medical assistance. At admission, she was febrile with absence of pulses on the right lower extremity and a diminished pulse on the left. She had a peripheral blood eosinophilia of 10%. Emergency femoral angiography indicated complete obstruction of the right common iliac and left internal iliac arteries (Figure) with collateral supplies via lumbar arteries. Surgical intervention demonstrated a white clot from aortic bifurcation down to the right common iliac artery and the superficial and deep femoral arteries, surrounded by necrotic tissue and enlarged inguinal nodes. Right femoral embolectomy and aortofemoral bypass were performed. A 10% KOH preparation of the clot and dissected nodes showed branching septate hyphae.

Cultures of these specimens grew *Pythium insidiosum*. Itraconazole solution, terbinafine, and therapeutic vaccines (*3*) were administered. Extended-spectrum antimicrobial agents were also given during her hospital stay to combat nosocomial infections. Progressive wound necrosis and gangrene of the limbs dictated multiple debridements and subsequent limb amputations and hip disarticulation. Her clinical course worsened, and she died 2 months after admission.

The second patient was a 15-year-old boy with β-thalassemia/hemoglobin E disease, 10 years after splenectomy. The patient received frequent blood transfusions and monthly intravenous deferoxamine for secondary hemochromatosis. Six days before admission to our hospital, necrotizing cellulitis of both legs, unresponsive to intravenous antimicrobial agents, was diagnosed at a local hospital. The patient reported frequent swimming in standing water in rice fields. He was febrile with a leukocyte count of 26,000/mm^3^ and was given broad-spectrum intravenous antimicrobial agents. Severity of the lesions demanded surgical debridement (Figure A1), which showed necrosis of skin and subcutaneous tissue but intact fasciae and muscles. Tissue pathology and fungal staining did not demonstrate the etiologic agent. Surgical tissue Gram's stain and culture were nonrevealing, but fungal culture grew *P. insidiosum* after a few days. Isolates demonstrated white submerged colonies and microscopically showed sparsely septate hyphae 4–10 μm in diameter. Boiled grass blades were transferred onto the agar with the growth and incubated at 37°C for 24 hours. The blades were then put in a container with dilute salt solution for 2 hours at 35°C. They were subsequently placed on a slide, and mobile biflagellate zoospores were seen. Serum antibodies to *P. insidiosum* by immunodiffusion were detected in both cases. Femoral angiography showed no abnormalities. Since therapeutic immunogens were not available during that period, a combination of supersaturated potassium iodide (SSKI), itraconazole, and terbinafine was initiated. SSKI was discontinued after a month because of side effects. Lesions were progressive involving fasciae and muscles and necessitated 2 episodes of surgical debridement during the first month. The lesions began to improve in the second month of medical therapy, when skin grafting was performed. The patient remained well and was discharged after almost 3 months. Medications were continued for a total period of 6 months. The patient has been well for >2 years.

*P. insidiosum* in tissues resembles agents of zygomycosis morphologically but, unlike the latter, rarely stains with hematoxylin and eosin. Various immunostainings also help identify the organism (*4*). With the exception of the facial-cranial form of the disease in the United States (*5*), most cases in Thailand occur in patients with chronic hemolytic anemia; thalassemia-hemoglobinopathy is the most common underlying disease. Major clinical manifestations include ocular and craniofacial infections in healthy children, arteritis usually originating from lower extremities, and chronic subcutaneous abscesses.

Treatment options reported to be successful include supersaturated potassium iodide for the chronic cutaneous form (*2*), a combination of terbinafine and itraconazole in a single case of an acute, severe ocular, subcutaneous infection (*6*), and therapeutic vaccination for severe infections involving major arteries (*7**,**8*) in conjunction with surgery. The first case suggested that, with chronic pythiosis involving the aorta, effective management is difficult. In the second case, the laboratory was familiar with *P. insidiosum* isolation procedures; therefore, a quick diagnosis was made and early treatment was instituted. This early form of cutaneous pythiosis is rarely diagnosed properly by most clinical and pathologic laboratories. Human cases probably occur worldwide (*9*) but are underrecognized and thus, misdiagnosed (*5*). More research into the pathogenesis, diagnosis, and new treatment modalities is urgently needed.

**Figure:**
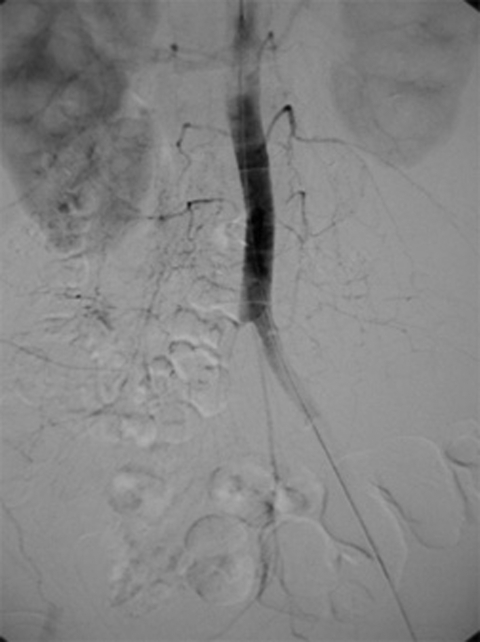
Femoral angiography of patient 1 demonstrating involvement of the right common iliac artery upward to the aorta and extending to the left internal iliac artery.

## Figures and Tables

**Figure A1:**
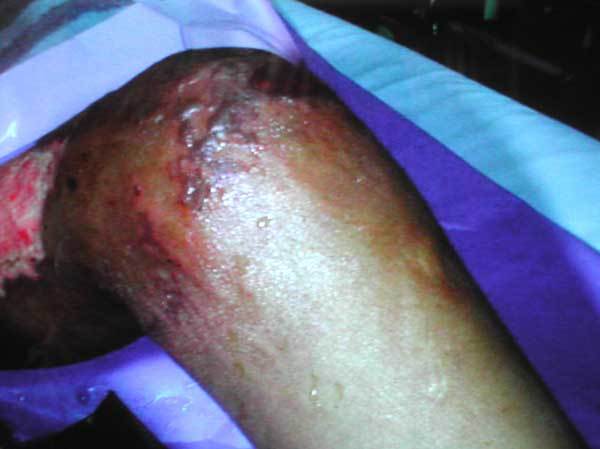
Surgically debrided lesions.
